# Novelty Improves the Formation and Persistence of Memory in a Naturalistic School Scenario

**DOI:** 10.3389/fpsyg.2020.00048

**Published:** 2020-01-29

**Authors:** D. Ramirez Butavand, I. Hirsch, M. Tomaiuolo, D. Moncada, H. Viola, F. Ballarini

**Affiliations:** ^1^Laboratorio de Neurociencia Traslacional, Instituto de Biología Celular y Neurociencias “Dr. Eduardo De Robertis” (IBCN), CONICET - Universidad de Buenos Aires, Buenos Aires, Argentina; ^2^Facultad de Medicina, Universidad de Buenos Aires, Buenos Aires, Argentina; ^3^Laboratorio de Memoria, Instituto de Biología Celular y Neurociencias “Dr. Eduardo De Robertis” (IBCN), CONICET - Universidad de Buenos Aires, Buenos Aires, Argentina; ^4^Instituto de Biología Celular y Neurociencias “Dr. Eduardo De Robertis” (IBCN), CONICET - Universidad de Buenos Aires, Buenos Aires, Argentina; ^5^Departamento de Fisiología, Biología Molecular y Celular “Dr. Héctor Maldonado” (FBMC), Facultad de Ciencias Exactas y Naturales, Universidad de Buenos Aires, Buenos Aires, Argentina

**Keywords:** long-term memory, high-school students, memory formation, memory persistence, novel class

## Abstract

One of the top challenges in education and neuroscience consists in translating laboratory results into strategies to improve learning and memory in teaching environments. In that sense, during the last two decades, researchers have discovered specific temporal windows around learning, during which the intervention with some experiences induces modulatory effects on the formation and/or persistence of memory. Based on these results, the aim of the present study was to design a specific strategy to improve the memory of students in a high-school scenario, by assessing the effect of a novel situation experienced close to learning. We found that the long-term memory about a geometrical figure was more precise in the group of students that faced a novel situation 1 h before or after learning the figure than the control group of students who did not face the novelty. This enhancement was probably triggered by processes acting on memory formation mechanisms that remained evident 45 days after learning, indicating that the improvement was sustained over time. In addition, our results showed that novelty no longer improved the memory if it was experienced 4 h before or after learning. However, far beyond this window of efficacy, when it was faced around 10 h after learning, the novel experience improved the memory persistence tested 7 days later. In summary, our findings characterized different temporal windows of the effectiveness of novelty acting on memory processing, providing a simple and inexpensive strategy that could be used to improve memory formation and persistence in high-school students.

## Introduction

Memory plays a central role in human life by shaping our identity, guiding our thoughts and decisions, and also by influencing our emotional reactions (Bisaz et al., 2014). For that reason, many researchers have focused on the study of the precise mechanisms involved in different aspects and phases of memory.

Lasting memories are indeed born labile and become stable through a process known as consolidation. However, consolidation is delicate and can be affected by experiences or different factors such as novelty, stress or arousal close to learning, all of which may modify the formation of a long-term memory (LTM) (McGaugh, 2000, 2004; Wittmann et al., 2005; Adcock et al., 2006; Schwabe et al., 2008; Roozendaal and McGaugh, 2011). During the last few decades, researchers have evaluated the behavioral and molecular mechanisms that occur around learning and provided some explanation for the fact that memories are consolidated. The first discoveries showed that memory consolidation relies on the availability of newly synthesized proteins required to induce plastic changes (Costa-Mattioli and Sonenberg, 2008), that are proposed to modify the connections between neurons in order to allow memory storage. Even, the processing of similar memories involves the activation of overlapping brain cell populations; whereas synapse-specific plasticity guarantees the identity and storage of individual memories (Abdou et al., 2018).

Reaching this level of specificity was originally studied in models of synaptic plasticity and was translated into behavior throughout the behavioral tagging (BT) hypothesis (Moncada and Viola, 2007). This hypothesis postulates that a population of cells tagged during learning can capture proteins synthesized either by the learning experience itself or by other events occurring within a critical time window. This conceptual framework allows us to understand how and when experiences, occurring close to a particular learning, can either have a positive or negative impact on the formation of lasting memories (for a review see: Moncada et al., 2015). In particular, the novel experiences promote the formation of aversive or spatial LTMs by inducing protein synthesis in the dorsal hippocampus of rats (Moncada and Viola, 2007; Wang et al., 2010). Moreover, the role of the dopaminergic neurotransmission in that process was identified as critical (Moncada et al., 2011; Moncada, 2017). Dopamine (DA) is released within the hippocampus from the ventral tegmental area to process novelty signal (Lisman and Grace, 2005). Also, recent optogenetics studies have suggested that the locus coeruleus may be another source of dopaminergic signaling associated with novelty-promoted hippocampal learning (Kempadoo et al., 2016; Takeuchi et al., 2016; McNamara and Dupret, 2017). The role of the substantia nigra/ventral tegmental area (SN/VTA), hippocampus and also DA in memory retention has been underlined in functional imaging studies in humans (Chowdhury et al., 2012; Bunzeck et al., 2014), and therefore, the hippocampal-VTA model helps to explain the beneficial effects of novelty on long-term memory.

Furthermore, the “life of a memory” is extremely dynamic. Thus, several processes, occurring in distant temporal windows after learning, are involved in regulating its persistence (Bekinschtein et al., 2007, 2008; Rossato et al., 2009; Katche et al., 2016) as well as keeping memory updated (Nader et al., 2000). In particular, Tomaiuolo et al. (2015) have demonstrated that animals exposed to a novel event 11 h after an aversive training showed an improvement in memory persistence, assessed 7 days after learning. This result was explained by a late tag of the memory, which is involved in the persistence of LTM storage.

Our hypothesis is that the BT is a conserved process that drives the formation of long-term memories and therefore human lasting memories are also established through it. In that sense, research in humans has already given its first steps with studies following this line of thought. Chowdhury et al. (2012) suggested that dopamine could rescue the memory of forgotten items by subsequent protein synthesis; whereas Dunsmoor et al. (2015) showed that strong experiences associated with weak learning improved its LTM expression. Finally, our group designed a series of experiments in elementary schools to evaluate how educational experiences (novel classes) close to a particular lesson may account for memory improvements for the content taught by their teachers (Ballarini et al., 2013).

Thus, the main objective of this research was to evaluate whether the effect of novelty on memory formation in elementary-school students could also be observed in high-school students and, at the same time, analyze the existence of a late window for novelty to modulate memory persistence. Here, we present data obtained in Argentinean high-schools characterizing selective time windows, in which a novel situation can improve memory formation and persistence.

## Methods

### Participants

The study involved a total of 490 participants (aged between 12 and 15 years old) from two different high schools in Buenos Aires, Argentina. All the students were naive to the procedure. This study was performed under the approval of the Ethical Committee of the Instituto de Tisioneumonología “Prof. Dr. Raúl Vaccarezza” of the University of Buenos Aires, Buenos Aires, Argentina. Procedures were reviewed and approved by the Head of each participating educational institution and a consent form was signed by students’ parents. Students were allowed to withdraw from the study at any time without consequences.

**Figure 1:**
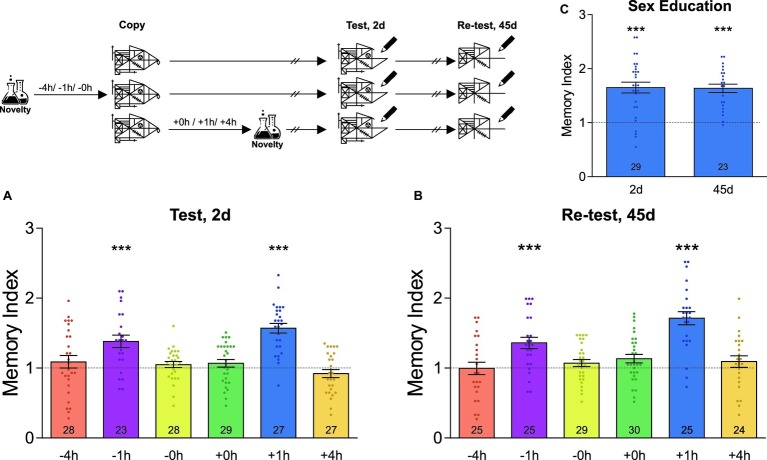
Novel experiences around learning improve the formation of an unrelated graphical memory. A schematic representation of the experimental protocol is presented on the top left of the figure: students were asked to copy Rey-Osterrieth’s figure and had or not (Control) a novel experience (Attentional Blindness class) before or after it. The time at which the students copied the figure was time zero and the time condition described for the novelty was relative to it and is expressed in hours. The Memory Index was calculated by normalizing the score obtained by each participant in the test session with its corresponding control group mean. It is shown as the mean ± SEM for students who have had the novel experience at different times around the time at which they copied the figure. The dotted line represents the Control group. **(A)** The LTM of this figure was tested 2 days after training. **(B)** The LTM of this figure was re-tested 45 days after training in the same students as those of figure **(A)**. **(C)** Students experienced a novel sex education lesson 1 h after training and tested 2 days after learning and re-tested 45 days later. One-sample *t*-test against theoretical value 1, ^***^p < 0.001. The number of students in the different experimental groups is detailed inside each bar. The number of students in all control groups ranged from 29 to 35.

**Figure 2:**
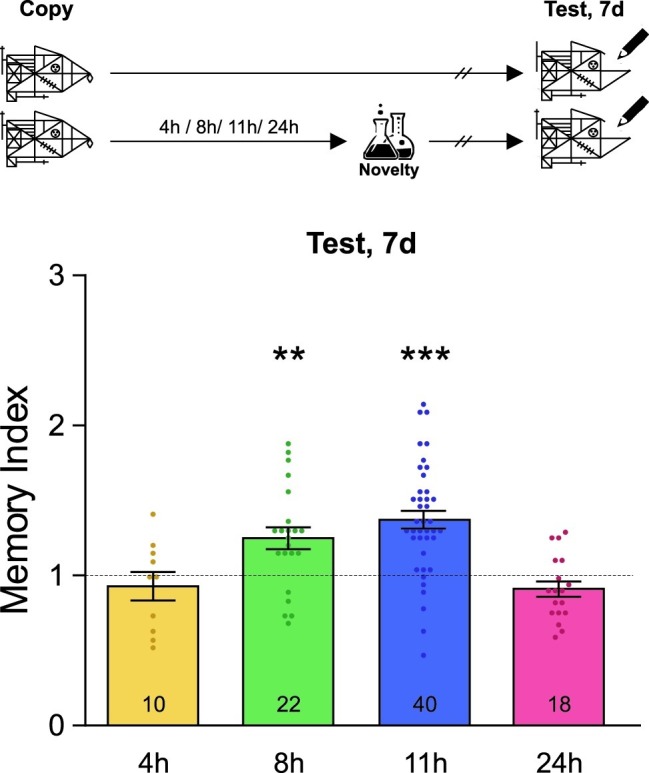
Novel experiences late after training improve memory persistence. A schematic representation of the experimental protocol is presented on the top panel: students were asked to copy Rey-Osterrieth’s figure and they had or not (Control) a novel experience (Attentional Blindness class) after it. The time at which the students copied the figure was time zero and the time condition described for the novelty was relative to it and is expressed in hours. The Memory Index was calculated by normalizing the score obtained by each participant in the test session with each corresponding control group mean. It is shown as mean ± SEM for students who have had the novel experience at different times after learning (4–24 h). The dotted line represents the Control group. The LTM shown in this figure was tested 7 days after training. One-sample *t*-test against theoretical value 1, ^**^p < 0.01, ^***^p < 0.001. The number of students in the different experimental groups is detailed inside each bar. The number of students in all control groups ranged from 30 to 37.

Figure 1 demonstrates the data composed from first-year students, i.e. the age range is 12–13 years old, while Figure 2 demonstrates the experiments performed on first-year students (in the four conditions) and on third-year students (8 and 11 h conditions; 14–15 years old).

We calculated that at least 15 participants per group were necessary in order to detect a testing effect of size *d* = 0.80 with a statistical power of 0.90 (alpha = 0.05). An efficient way to achieve this was to assign an entire school course per group, which in fact usually exceeded this minimum required threshold.

### Procedures

A graphic memory study was carried out. Briefly, the first day (Training Day) students were shown Rey-Osterrieth’s complex figure and provided with 5 min to copy it (Control). In parallel, another group of students from the same institution and of the same age as the control group was shown this figure and also had the same time to copy it, but, this time, the training was associated with a novel lesson (Attentional Blindness class) either before or after it. We worked with two different paradigms, i.e., the effect of the novel experience was to be evaluated with regards to separate aspects of memory: formation and persistence. For the former, first-year students experienced the novel lesson either immediately, 1 or 4 h before or after copying the figure. LTM was then evaluated anonymously in each group 2 and 45 days later. The students were asked to draw the figure again on a blank paper in 4 min. To evaluate memory persistence, the students experienced the novel lesson either 4, 8, 11 or 24 h after copying the figure, and LTM was evaluated 7 days later as described above.

In another pair of groups, we assessed the effect of a different novel lesson (Sex education class) experienced 1 h after training and then tested the graphic memory 2 and 45 days later.

### Novel Lessons

For an activity to be considered as a novelty, it had to comply with the following requirements: (1) the whole group of students was unexpectedly taken from their classroom and led to a different place to attend a lesson that was not previously informed about until it started; (2) this novel lesson was given in a place inside the school but not usually frequented by students for their lessons; (3) the lesson was given by a skilled teacher, unknown to the students; (4) it was a short activity (20 min), never before experienced by the students, with novel contents, appropriate for their age; (5) students were encouraged to actively participate and be attentive at all times. When the activity finished, they returned to their habitual classroom.

The Attentional Blindness class was based on a science class, containing simple experiments, aimed to the constant participation of the students and their full interaction with the elements. During the lesson, they were invited to play some games, to illustrate how people think that they can pay much more attention than they actually do. Specifically, we developed two main topics: change blindness (a phenomenon in which a very large change in a picture will not be seen by a viewer) using pictures from http://nivea.psycho.univ-paris5.fr/ and inattentional blindness (phenomenon in which a participant is looking at a video sequence and their attention is so captured by the task he/she is doing that something totally obvious is not noticed), using videos from http://www.theinvisiblegorilla.com. Afterwards, some basic biological and neuroscientific principles were explained to the students to provide them with some theoretical background on the experiments. It is worth noting that throughout the period of a high-school education, it is unusual for students to be involved in an activity that requires them to pay attention and then not be tested in that specific topic.

In the sex education class, the researcher addressed some basic principles of reproduction, safety and prevention of sexually transmitted diseases, but also giving the students space to ask any questions they had on the matter, creating a group debate on the subject. This topic was selected due to its appeal to teenage students. Besides that, it provided the opportunity of giving some primary prevention advice in an environment where they could speak freely with people they did not know before; thus helping them to be spared from embarrassment typically found in that type of situation.

### Data and Statistical Analysis

To quantify the memory performance, the location, accuracy and organization of the items that integrated Rey-Osterrieth’s figure were analyzed according to a scoring scheme (Rey, 1959). The Memory Index for each student was calculated by normalizing the score obtained in the test session to the mean of their own control group. Results were presented as mean ± SEM. Data was analyzed using One-sample *t*-test against theoretical value 1. A result was considered significant when *p* < 0.05. All data was analyzed using GraphPad Prism® software.

## Results

Using cognitive tests based on Rey-Osterrieth’s figure, we first evaluated the consequences of undergoing a novel experience in the school environment on the formation of a graphical memory. In order to do that, the high school students were told to copy the figure during a regular class session. In some cases, students from different classes also attended a novel laboratory interactive lesson (not included in their regular curricula) either immediately, 1 or 4 h before or after copying the figure. Memory was assessed 2 days later by asking the students to draw whatever they could remember of the original figure during class hours. As shown in Figure 1A, the groups of students that attended the novel science lesson 1 h before or after copying Rey-Osterrieth’s figure expressed a better memory than their control groups, composed of students who did not attend the novel lesson (one-sample *t*-test against theoretical value 1, −1 h: *t* = 4.26, *p* < 0.001; +1 h: *t* = 8.31, *p* < 0.001). In contrast, attending the novel class 4 h before or after copying Rey-Osterrieth’s figure, or immediately before or after, did not affect LTM (one-sample *t*-test against theoretical value 1, *p* > 0.05, Figure 1A). To further evaluate whether the improvements on LTM remained stable for long-lasting periods of time, a new test session was performed on the same students 45 days after training. As expected, in all the groups studied, this remote memory decreased between 30 and 40% compared to that recorded 2 days after training (Mean of the Memory Index on day 45 relative to day 2, −4 h: 0.64; −1 h: 0.69; −0 h: 0.64; +0 h: 0.66; +1 h: 0.77; +4 h: 0.84; in all cases, Student’s *t*-test revealed *p* < 0.05). Despite this, Figure 1B shows that the memory indexes in students who experienced the novelty 1 h before or after the training were significantly higher than those of the control group (one-sample *t*-test against theoretical value 1, −1 h: *t* = 4.42, *p* < 0.001; +1 h: *t* = 7.65, *p* < 0.001; any other schedule: *p* > 0.05).

To further analyze whether different novel experiences can impact students’ LTM, we next studied whether a novel sex education class was also able to improve the LTM of Rey-Osterrieth’s figure. As observed in Figure 1C, the students that attended this class 1 h after learning the Rey-Osterrieth’s figure showed 65% of enhancement in 2-day LTM with respect to the control group (one-sample *t*-test against theoretical value 1, *t* = 6.56, *p* < 0.001). This improvement was maintained for at least the next 45 days (one-sample *t*-test against theoretical value 1, *t* = 8.11, *p* < 0.001). This result indicates that improvement in memory is independent of the type of novelty and depends exclusively on the time window around the learning in which it occurs.

Finally, we decided to analyze whether novel lessons experienced far away from Rey-Osterrieth’s figure learning could improve the persistence of this memory. In order to do this, different groups of students either attended or did not attend a novel attentional blindness science class 4, 8, 11, or 24 h after learning Rey-Osterrieth’s figure and their memory was evaluated 7 days later. As shown in Figure 2, novelty improved this LTM when it was experienced in a late and restricted time window between 8 and 11 h after learning (one-sample *t*-test against theoretical value 1, +8 h: *t* = 3.38, *p* = 0.0028; +11 h: *t* = 6.25, *p* < 0.001). In contrast, no effect was seen when students experienced the novel class 4 or 24 h after the training (one-sample *t*-test against theoretical value 1, *p* > 0.05). These results corroborate the existence of a late maintenance-memory phase and show that, within it, novelty can affect the persistence of LTM about graphical information learned in class.

## Discussion

Here, we showed that within a specific time window, novel lessons could improve the memory of the content learned during class hours. Specifically, attending a novel sex education or attentional blindness class 1 h before or after learning Rey-Osterrieth’s figure improved this graphical LTM in a lasting way, as recorded 2 and 45 days after learning. Moreover, we showed that when the novel attentional blindness class was experienced 8 or 11 h, but not 24 h, after Rey-Osterrieth’s figure learning, LTM persistence improved. However, when the novel class was attended 4 h after learning, the students’ memory score did not improve either at 2 or 7 days after training. These results suggest that the existence of a specific memory formation time-window (1 h around learning, before or after it), different from the memory persistence temporal window (around 10 h after learning), confirming previous results in rodents (Moncada et al., 2015). Therefore, the formation and persistence of the information learned in the classroom can be modulated by other experiences, such as novel lessons, highlighting the importance of the temporal course of its application to affect different phases of memory.

This late association between stimuli and events may be explained by the synaptic tagging and capture hypothesis (Frey and Morris, 1997) and its behavioral counterpart, the BT hypothesis (Moncada and Viola, 2007). This hypothesis postulates that tags set in sites activated during learning are capable of capturing newly synthesized proteins to allow the consolidation process and memory storage. Therefore, the cellular interactions are limited to events processed in a shared neuronal population as well as by the tags duration and the dynamics of protein synthesis and degradation (Moncada et al., 2015). In that sense, as far as they remain stable, tags set upon a learning event can make use of proteins synthesized by a later experience, allowing or improving memory formation. In the inverse order, previously synthesized proteins can be captured by tags set in a later learning event. On the other hand, when two events occur too close in time (novelty experienced immediately before or after learning), alterations in their tags may affect the resulting memory, impairing its enhancement. Thus, a possible explanation for our results is that the molecular processes triggered by the learning and the novel class could interact within a temporary time-window, while too close or to broader intervals could prevent this interaction, suggesting that a BT process may underlie the formation of this memory.

These results are in agreement with our previous findings, obtained in elementary schools, showing that novel experiences occurring 1 h but not 4 h before or after a storytelling experience were able to improve a literary LTM (Ballarini et al., 2013). They are also in line with our recent observations regarding mild stress, associated with an exam, which can improve the graphical LTM in this time window (Lopes da Cunha et al., 2018). In addition, here we describe a narrow temporal window very close to the learning moment (immediately before or after it), in which novel experiences were ineffective in enhancing the graphical LTM. It is also worth noting the remarkable similarity between this time window and that observed in rodents during the behavioral tagging studies that inspired this research (Ballarini et al., 2009). In that sense, different laboratories have demonstrated that novelty is able to promote the formation of otherwise inexistent LTMs when it is experienced within a critical time window comprising the hour around training, but excluding time points beyond 2 h and immediately before or after the learning session (Redondo and Morris, 2011; Moncada et al., 2014; Vishnoi et al., 2016). Thus, the whole body of evidence suggests that the 1-h interval before or after learning is ideal to associate novel experiences to enhance the memory of content recently learned in the classroom.

Further analysis showed that the memory improvement induced by the novelty 2 days after learning was sustained for more than a month. Therefore, it is possible that the first test could be, at least in part, responsible for this long-lasting durability. Indeed, this is in agreement with the phenomenon of *Test-enhance learning*, referring to the improvement of memories when teaching is accompanied with an evaluation (Roediger et al., 2011). In addition, this memory maintenance can be also analyzed under the mechanisms of memory reconsolidation (Nader et al., 2000). It is possible that the 2-day test would act as a reminder, inducing memory labilization and activating the cellular processes that allow the memory to be updated and better preserved for longer periods (Lee et al., 2017). In any case, it is worth noting that the improvement induced by novelty keeps sustained during these 45 days, regardless of the mechanism responsible for maintaining the long lasting-memory. This outcome is very useful in the school context where most of the topics learned are tested several weeks later.

Recently, experiments performed in rodents have shown the existence of a late memory phase acting around 10–12 h after training to specifically regulate memory persistence (Bekinschtein et al., 2007; Tomaiuolo et al., 2015; Katche et al., 2016). Different interventions at that time window can establish, improve or prevent the expression of the persistent LTM (defined by being tested 1 week after learning). For the first time, it becomes clear that a similar process also occurs in humans. In this work, we observed that attending a novel class around 10 h after learning Rey-Osterrieth’s figure improved the graphic memory 1 week later. This time point goes beyond the 4 h in which the novelty is no longer able to improve memory formation (Figure 1 and Ballarini et al., 2013). Thus, our results support the existence of a late phase of memory storage, in which novel events can modulate the persistent memory about contents learned during class hours.

In conclusion, it seems that there are two different moments at which novel experiences can modulate memory formation and persistence. The present results can be explained by a broader version of the BT hypothesis, which proposes that the durability of a memory trace depends on a learning-tag mechanism working close to the acquisition, and also on a maintenance-tag mechanism operating several hours after training (Moncada et al., 2015). In a wider scenario, it should be noted that, in opposition to the improving effects of novelty, other experiences are able to affect the tags or interfere with the availability of proteins in the different time windows might result in memory impairments. Therefore, an important behavioral implication of our findings relies on the fact that the possibility to modulate the improvement of LTM depends not only on events occurring relatively close to the moment of learning, but also on other events occurring late after it.

The scientific advances in cognitive neuroscience inspired us to search for behavioral interventions able to provide benefits to the learning process at school. Our findings support those previously obtained in laboratory research animals, highlighting the degree of conservation in memory processing across nature. In addition, they contribute to the educational process by offering simple strategies to use at schools to improve memory formation and persistence.

## Data Availability Statement

The raw data supporting the conclusions of this manuscript will be made available by the authors, without undue reservation, to any qualified researcher.

## Ethics Statement

The studies involving human participants were reviewed and approved by Ethical Committee of the University of Buenos Aires Instituto de Tisioneumonología “Prof. Dr. Raúl Vaccarezza”. Written informed consent to participate in this study was provided by the participants’ legal guardian/next of kin.

## Author Contributions

DR, IH, MT, and FB conducted the activities at school and analyzed the data. HV, DM, MT, and FB designed the study. DR, DM, HV, and FB wrote the manuscript. All the authors contributed to revising and reading the manuscript and approved the submitted version.

### Conflict of Interest

The authors declare that the research was conducted in the absence of any commercial or financial relationships that could be construed as a potential conflict of interest.
